# Full-Length Transcriptome Sequencing and hsp Gene Family Analysis Provide New Insights into the Stress Response Mechanisms of *Mystus guttatus*

**DOI:** 10.3390/biology14070840

**Published:** 2025-07-10

**Authors:** Lang Qin, Xueling Zhang, Yusen Li, Jun Shi, Yu Li, Yaoquan Han, Hui Luo, Dapeng Wang, Yong Lin, Hua Ye

**Affiliations:** 1 Key Laboratory of Freshwater Fish Reproduction and Development, Ministry of Education, College of Fisheries, Southwest University, Chongqing 402460, China; qinlang040126@163.com (L.Q.); 15281849319@163.com (X.Z.); ly085020@126.com (Y.L.); luohui2629@vip.163.com (H.L.); 2 Yibin Academy of Southwest University, Yibin 644005, China; 3 Fisheries Research Institute, Sichuan Academy of Agricultural Sciences (Sichuan Fisheries Research Institute), Chengdu 611731, China; 4 Guangxi Key Laboratory of Aquatic Genetic Breeding and Healthy Aquaculture, Guangxi Aquatic Breeding Base, Guangxi Academy of Fisheries Science, Nanning 530021, China; liyusen@mail2.sysu.edu.cn (Y.L.); shijun19720308@sina.com (J.S.); hyqao@sina.com (Y.H.); oucwdp@163.com (D.W.)

**Keywords:** full-length transcriptome, stress response mechanisms, heat shock protein, *Mystus guttatus*

## Abstract

*Mystus guttatus*, a second-class protected species in China, has undergone severe population decline due to anthropogenic and environmental pressures, yet conservation efforts are hindered by limited genomic resources and a lack of mechanistic insights into its stress response systems. Here, the first full-length transcriptome of *M. guttatus* was generated via SMRT sequencing. A total of 32,647 full-length transcripts were obtained, with an average length of 1783 bp. After structure and function annotation of full-length transcripts, 30,977 genes, 1670 transcription factors (TF), 918 alternative splicing (AS), and 11,830 simple sequence repeats (SSR) were identified. In order to further explore the stress resistance of *M. guttatus*, 93 genes belonging to the heat shock protein (HSP) family were identified and categorized into HSP70 and HSP90 subgroups. After phylogenetic analysis and selective stress analysis, it was discovered that the *hsp* family has suffered purifying selection and gene loss, potentially contributing to a decrease in the stress resilience and population of *M. guttatus.* Using protein interaction network and molecular docking tools, we observed the intricate interplay among HSPs and discovered HSP70-HOP-HSP90 interaction, which is an essential stress response mechanism. Our study sequenced the first full-length transcriptome of *M. guttatus* to enhance its genomic resources for its conservation and breeding and provide new insights into the future study of stress response mechanisms on *M. guttatus*.

## Simple Summary

*Mystus guttatus*, a rare and protected fish in China, has seen its population plummet due to human activities and environmental changes. To aid its conservation, scientists studied its stress response mechanisms using advanced gene sequencing SMRT. They generated the first full-length “transcriptome” (all functional genes) of this fish, revealing 32,647 genes. In order to further explore the reasons for the decline in the population of *M. guttatus* at the genomic level, their focus was on heat shock proteins (HSPs), which help organisms cope with stress like temperature changes. The team found 93 HSP genes, split into two groups (HSP70 and HSP90). These genes had signs of “purifying selection” (natural pruning of less useful versions) and some gene loss, possibly weakening their stress resistance over time. They also discovered HSPs work together in a network (HSP70-HOP-HSP90 complex) to manage stress. This info provides new tools for breeding programs and highlights how historical genetic changes might make the fish vulnerable today. Understanding these mechanisms can guide efforts to boost their resilience and aid recovery.

## 1. Introduction

**Figure 1:**
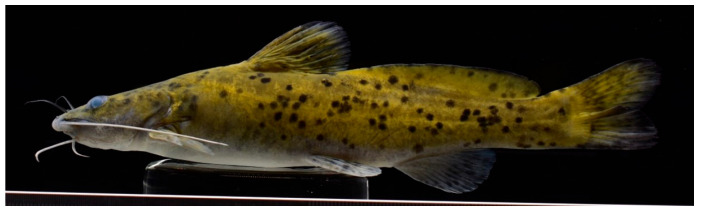
Picture of adult *Mystus guttatus*.

*Mystus guttatus*, classified within the class Siluriformes, family Bagridae, and genus *Mystus* (Figure 1), is primarily distributed in China’s Pearl River and Xijiang River [1]. *M. guttatus* has been regarded as one of the most valuable economic species in the Pearl River, attributable to its flavorful and nutritious meat [2]. However, anthropogenic disturbances, including dam construction, industrial effluent discharge, overexploitation, and habitat degradation, have precipitated severe population declines in recent decades [3,4,5]. In 2021, *M. guttatus* was listed as a second-class protected species in China’s List of Key Protected Wild Animals [6]. Previous research on *M. guttatus* has mainly focused on gonadal development [7], artificial reproduction [8], and artificial breeding [9], aiming to carry out breeding programs for its resource conservation. Although these studies have achieved significant results, persistent challenges in juvenile survival rates continue to impede population recovery initiatives [10].

Stress resilience constitutes a critical survival determinant for aquatic organisms facing environmental challenges [11]. The heat shock protein (HSP) superfamily, including the HSP100, HSP90, HSP70, HSP60, and HSP40 subfamilies, plays pivotal roles in cellular stress adaptation by maintaining proteotoxic under environmental perturbations [12]. Beyond their canonical roles in protein folding dynamics, those proteins serve as valuable models for evolutionary studies due to their ubiquitous taxonomic distribution [13]. Systematic characterization of HSP family members could therefore elucidate stress response mechanisms in *M. guttatus* while informing strategies for genetic improvement [14]. However, research in stress tolerance and the HSP family of *M. guttatus* remains constrained by insufficient genomic resources [15,16,17].

Single-molecule real-time (SMRT) sequencing, a third-generation sequencing technology, facilitates direct acquisition of full-length transcript isoforms without assembly requirements [18]. This methodology serves as a robust platform for developing comprehensive functional genomic resources and polymorphic genetic markers in non-model species [19]. Recent advances in genomic research have demonstrated the successful implementation of SMRT sequencing across diverse taxa for critical applications including gene structure characterization, alternative splicing (AS) profiling, transcription factor (TF) identification, and simple sequence repeat (SSR) discovery [20,21,22,23].

Here, we present the first full-length transcriptome of *M. guttatus* generated via SMRT sequencing. We comprehensively annotated functional elements, including coding genes, TFs, AS variants, and SSRs, with particular emphasis on HSP superfamily characterization. Through integrative analyses of phylogenetic relationships, physicochemical properties, subcellular localization, protein interaction networks, and molecular docking, we delineated the structural and functional landscape of the HSP70 and HSP90 subfamilies. Evolutionary dynamics were further explored through gene loss and purifying selection analyses. This study establishes foundational genomic resources for *M. guttatus* conservation and breeding programs while providing novel insights into stress adaptation mechanisms in this threatened species.

## 2. Materials and Methods

### 2.1. Sample Collection

The experimental fish was captured in Laibin, Guangxi Province with a body length of 32.34 cm and a weight of 283.8 g. Nine tissue samples, respectively, from its liver, intestine, gonad, kidney, muscle, skin, adipose fin, gill, and brain, were collected after anesthesia with 40 mg/L eugenol. All the samples were preserved in RNAlater at −80 °C for RNA extraction. All operations of this experiment were performed in compliance with the Animal Management Regulations of the Animal Welfare and Ethical Committee of Southwest University (Chongqing, China).

### 2.2. Total RNA Extraction

The total RNA of each tissue was extracted using the TaKaRa MiniBEST Universal RNA Extraction Kit (Takara, Dalian, China). The quality of RNA was detected by 1% agarose gel electrophoresis, and the concentration and purity of DNA were further detected by NanoDROP 8000 (Thermo Fisher, Waltham, MA, USA). The RNAs from all tissues were pooled in equal amounts and stored at −80 °C for later use.

### 2.3. PacBio Library Construction and Sequencing

Following the enrichment of qualified RNA for mRNA using oligo (dT), cDNA was synthesized through PCR amplification. The genome was then fragmented, and the ends of the fragmented DNA were repaired. Single-stranded hairpin-like junction sequences were added at both ends to construct the SMRTbell library. The size of the DNA fragments was carefully controlled during the library construction process to meet the requirements of the PacBio sequencing platform. Finally, load the constructed library into the PacBio sequencer for single-molecule real-time sequencing.

### 2.4. SMRT Sequencing Data Processing

The raw data generated by Sequel2 were analyzed using SMRT Link V8.0 [24]. Firstly, Circular Consensus Sequencing (CCS) reads, with at least one full pass, were extracted from the sequencing data. The extracted reads, with the 5′ primer, the 3′ primer, and the poly-A structure, were further filtered to obtain the full-length non-chimeric sequence (FLNC). The similar FLNC reads were hierarchically clustered to obtain Unpolished Consensus Isoforms (UCIs). Then, the UCIs were corrected by the Quiver algorithm, to produce high-quality sequences (HQ isoforms) with a prediction accuracy of at least 99%. The HQ isoforms were de-redundant using the cd-hit-v4.6.7 software, and sequences with a similarity above 99% were merged [25]. Full-length transcript sequences were obtained using the local alignment method. For short sequences, the alignment rate must reach 99%, and the number of unmatched bases must be less than 30 bp. Alignment criteria included an alignment rate of 99% and a maximum of 30 bp for short sequences, and an alignment rate of 90% with a maximum of 100 bp for long sequences.

### 2.5. Full-Length Transcriptome Annotations Analysis

To gain a complete understanding of gene function information, isoforms were annotated in the Nr, Nt, KOG, Swiss-Prot, and UniProt databases. The coding sequences were conducted by Cogent [26]. The AS was analyzed with SUPPA [27], and they were categorized as Skipping Exon (SE), Alternative 5′ Splice Site (A5), Alternative 3′ Splice Site (A3), Retained Intron (RI), Alternative Last Exon (AL), and Alternative First Exon (AF). With hmmscan, the TF was determined in TF databases (like plant TFdb and animal TFdb). The SSRs of full-length transcriptome were identified using MISA v1.0 (http://pgrc.ipk-gatersleben.de/misa/misa.html (accessed on 20 December 2024)). Repeat units, with six dinucleotide repeats, five trinucleotide repeats, or four tetranucleotide, pentanucleotide, and hexanucleotide repeats, were recognized as SSRs. Additionally, SSRs with a distance shorter than 100 bp were merged into one, while those with sequence lengths less than 1000 bp were removed. The final number of SSRs was obtained from post-statistical analyses.

### 2.6. Basic Information About the HSP Family

To explore the stress response mechanism, HSP sequences were obtained from the annotation results. The molecular weight (Mw) and isoelectric point (Pi) of the amino acid sequences were estimated using Protparam 1.8 (https://web.expasy.org/protparam/ (accessed on 20 December 2024)). Wolfposrt 1.0 (https://wolfpsort.hgc.jp/ (accessed on 20 December 2024) and NetOGlyc 4.0 Server (http://www.cbs.dtu.dk/services/NetOGlyc/ (accessed on 20 December 2024)) predicted subcellular localization and glycosylation sites, respectively.

### 2.7. Interaction Mechanism Analysis of the HSP Family

All the gene sequences belonging to the HSP gene family were proofread, and STRING v2.0 (https://cn.string-db.org/ (accessed on 20 December 2024)) software was used to establish the protein interaction network of the HSP family. We set the parameter to a high confidence level (0.700), and unconnected protein members hidden in the network [28]. Then, the highly matched HSP70, HSP90, HOP, and GR were selected for molecular docking. Homology modeling was performed based on their sequences by Swiss-Model v1.0 (https://swissmodel.expasy.org/ (accessed on 10 December 2024)). HDOCK v1.0 (http://hdock.phys.hust.edu.cn/ (accessed on 10 December 2024)) was employed in molecular docking. PyMol 2.4 was used to model preprocessing, including removing water molecules and redundant ligands, as well as adding hydrogen atoms [29]. Docking Score, Confidence Score, and Ligand RMSD served as evaluation criteria for docking. A Docking Score greater than 200 indicates a certain level of binding affinity, 250 signifies good binding efficacy, and 300 represents the optimal binding affinity. Finally, the model with the highest score was selected as the optimal docking model and visualized.

### 2.8. Evolutionary and Motif Analysis of hsp Gene Family

The *hsp* family data from other Osteichthyes was downloaded in NCBI. They were combined with the *hsp* of *M. guttatus* for phylogenetic analysis. Multiple sequence comparison was performed using MAFFT v7.47 [30]. Then, the maximum likelihood tree was constructed using IQ-TREE v2.1.2 software, with a bootstrap value set to 1000 and other parameters default [31]. In addition, the sequence of highly matched proteins was preprocessed using the paraAT to ensure they were of the same length [32]. Subsequently, the KaKs_calculator software v1.5 was employed to calculate the Ka/Ks values in the *hsp* family of *M. guttatus* [33]. At the same time, conserved motifs in the amino acid sequences were analyzed using the MEME software v1.0 (http://meme-suite.org/ (accessed on 2 December 2024)), with a maximum number of recognizable motifs set to 6. Finally, TBtools software v2.0 was used to visualize the conserved motifs of HSP proteins [34].

## 3. Results

### 3.1. Full-Length Transcriptome Sequencing Data

**Figure 2:**
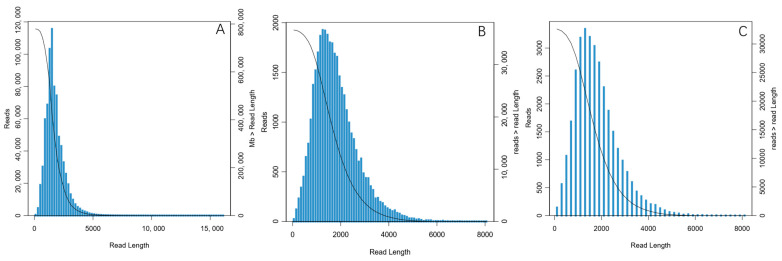
Length distribution of PacBio SMRT sequencing. (**A**) CCS read; (**B**) FLNC; (**C**) Single gene (The left vertical coordinate indicates the number of sequences of that length, and the right vertical coordinate indicates the number of sequences with a length greater than a certain value (x-axis).).

With PacBio SMRT sequencing technology, 63.4 G data were obtained. After filtering, a total of 779,206 high-precision CCS reads were detected (Figure 2A). Subsequently, FLNCs were screened from these CCS sequences. These FLNCs were then clustered and corrected, resulting in 36,338 high-quantity sequences (HQ isoforms) (Figure 2B). Finally, 32,647 full-length transcripts were generated for subsequent analysis after de-redundant processing (Figure 2C and Table 1).

### 3.2. Annotation and Analysis of Full-Length Transcriptome

**Figure 3:**
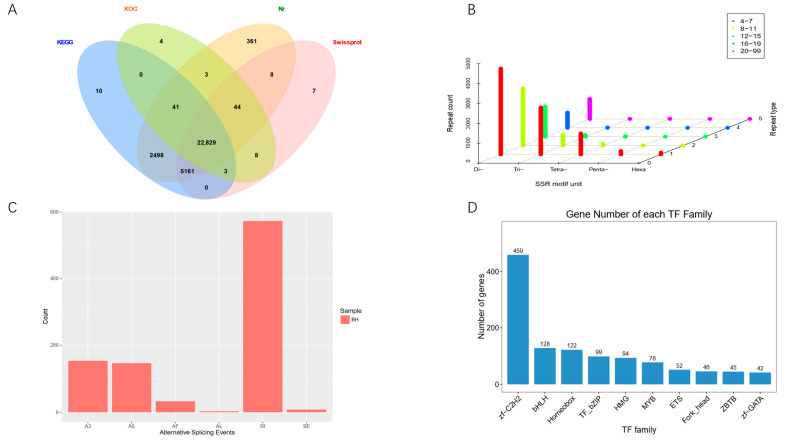
Annotation and analysis of full-length transcriptome. (**A**) Annotation status by database; (**B**) SSR distribution map; (**C**) Alternative splicing statistics; (**D**) Top 10 TF families.

Among 32,647 isoforms, 30,977 isoforms (94.9%) were annotated in at least one database, with 22,829 transcripts annotated by all four databases (Figure 3A). Nr, KEGG, KOG, and Swiss-Prot, respectively, annotated 30,945, 30,542, 22,932, and 28,060 transcripts. A total of 11,830 SSRs were identified in the sequences of 32,591 transcripts. Among the five types of SSRs, dinucleotide (P2) repeat sequences accounted for the highest proportion, followed by trinucleotide repeat sequences (P3) and tetranucleotide repeat sequences (P4). Additionally, the proportion of 4–7 repetitions was the highest within the SSRs (Figure 3B). Furthermore, a total of 918 AS events were detected in the transcripts of *M. guttuas* based on Iso-Seq reads. The distribution of these events was as follows: RI (62.42%), A3 (16.78%), A5 (16.01%), AF (3.59%), SE (0.87%), and AL (0.33%) (Figure 3C). In our study, 1669 TFs were identified, belonging to 57 different TF families, among which the top 3 were zf-C2H2, bHLH, and Homeobox (Figure 3D).

### 3.3. HSP70 and HSP90 Identification and Subcellular Localization

To investigate stress-responsive molecular mechanisms in *M. guttatus*, we performed functional annotation of the HSP superfamily using the Pfam database. This analysis identified 63 HSP70 and 30 HSP90 family members, with protein lengths spanning 205–1067 amino acids (AA). Subcellular localization predictions revealed distinct patterns: 57 HSPs were cytoplasm-localized, 17 localized to the nucleus, and 8 targeted to mitochondria. Notably, glycosylation site profiling demonstrated limited post-translational modifications, with most HSPs containing ≤ 1 glycosylation site. A striking exception was *MgHsp70-50*, which harbored five glycosylation sites—a feature potentially linked to enhanced stress-responsive regulation (Table S1).

### 3.4. HSP Family Phylogeny and Motif Analysis

**Figure 4:**
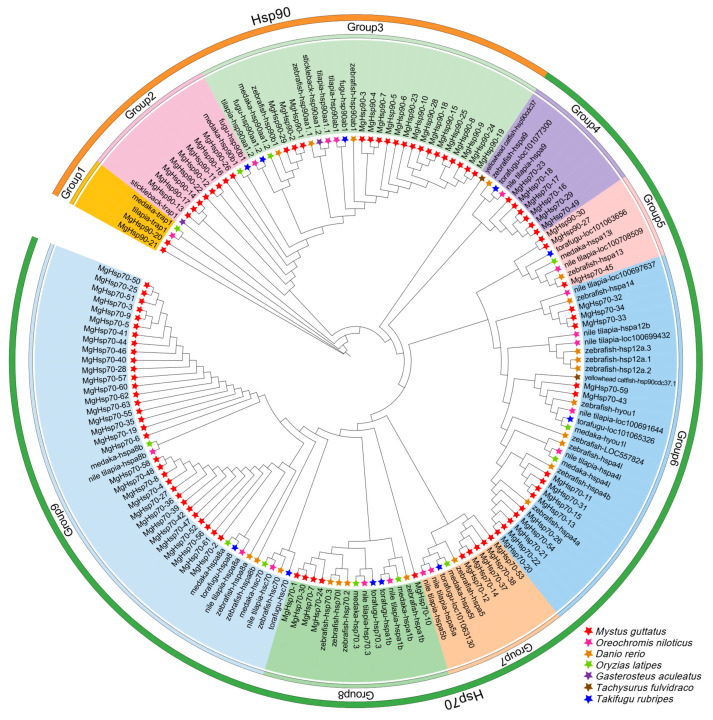
Phylogenetic maximum likelihood tree of the *hsp* gene family in fish.

**Figure 5:**
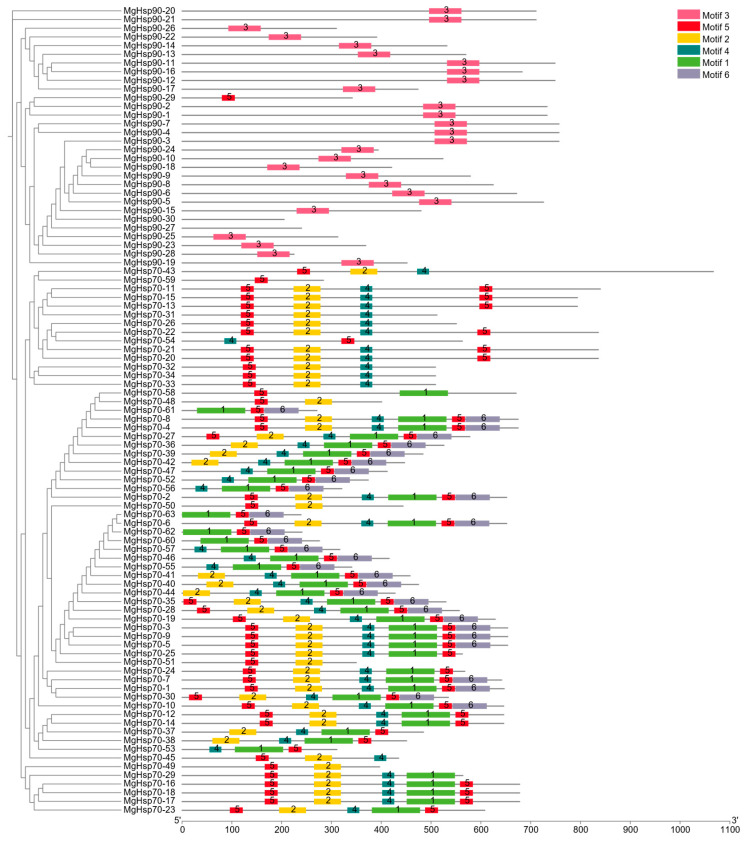
Motif analysis of *hsp* gene family.

To elucidate the evolutionary dynamics of the *hsp* family in fish, 5 species were selected as outgroups, resulting in a total of 120 genes, including *Oreochromis niloticus*, *Danio rerio*, *Oryzias latipes*, *Gasterosteus aculeatus*, *Tachysurus fulvidraco*, and *Takifugu rubripes*, to construct a maximum likelihood tree, where the proteins are divided into two main branches and nine groups. The HSP90 accounts for three groups, and HSP70 accounts for six groups (Figure 4). In motif analysis, the HSP family is divided into three groups, with only one motif in the HSP90 group, and all groups in the HSP70 group have motif 5 (Figure 5). Additionally, the HSP70 group was subdivided into two subgroups based on the presence or absence of motif 1, HSP70A (without motif 1) and HSP70B (with motif 1).

### 3.5. Interaction Mechanism Analysis of HSP Family

**Figure 6:**
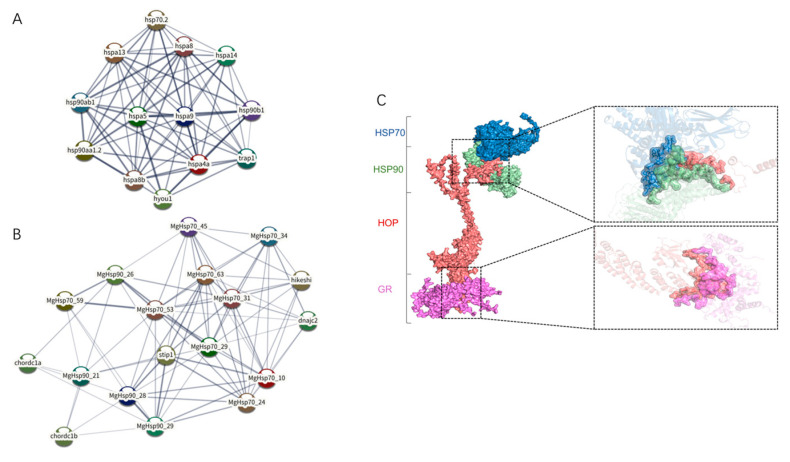
Interaction mechanism analysis of HSP70 and HSP90. (**A**) Ninety-three HSPs interact with each other; (**B**) The interaction network of 93 HSPs introducing other proteins; (**C**) Molecular docking of HSP.

To explore the potential interactions between HSPs, protein interaction networks were predicted. Then, it was found that all 93 HSPs were mapped onto the protein interaction network map (Figure 6A). Among the protein–protein interactions, most of the HSP interacted with heat shock protein organizing protein (HOP) (Figure 6B). Then, molecular docking was used to analyze the process of interaction of HSP70 and HSP90 (Figure 6C). Molecular docking was categorized into three groups: HSP70-HSP90, HOP-GR, and HSP70-HSP90-HOP-GR. Docking simulations were performed separately for the HSP70-HSP90 and HOP-GR pairs. After establishing docking models for these two groups, a composite docking simulation was conducted for the HSP70-HSP90-HOP-GR complex. Docking Score, Confidence Score, and Ligand RMSD were employed as evaluation metrics. Docking Score determines the binding efficacy between two proteins, where a higher absolute value indicates stronger binding. Generally, scores > 200 suggest moderate binding, >250 indicate strong binding, and >300 represent optimal binding [35]. Confidence Score and Ligand RMSD reflect confidence level and deviation, respectively (Table 2). All DOCKING SCORES of molecular docking results achieved exceeding 250. Specific binding interfaces for these protein pairs are detailed in Table S2. The “Receptor Interface Residue” column includes three subcolumns: receptor protein amino acid names, numbers, and chain identifiers. The subsequent “Hydrogen Bonds (Å)” column lists hydrogen bond lengths (Å) for these residues. Similarly, the “Ligand interface residue” column contains three subcolumns: ligand protein amino acid names, numbers, and chain identifiers, followed by hydrogen bond lengths (Å). The “Receptor-ligand interface residue pair(s)” column comprises five subcolumns: receptor amino acid number and chain identifier, a hyphen (“-”), ligand amino acid number and chain identifier, and hydrogen bond length (Å) between the residue pair.

### 3.6. Selective Pressure Analysis

To validate the findings from the phylogenetic analyses, selective pressure analyses were performed. Thirteen *hsp* genes, which have a high matched rate with zebrafish *hsp* genes, were selected to assess the selection pressure. The Ka/Ks rate of these 13 *hsp* genes was computed (Table 3). Twelve of the thirteen genes were under purified selection, with only HSP70-4 showing positive selection.

## 4. Discussion

As a rare economic fish and nationally protected grade II species with high research value, *M. guttatus* has faced challenges in conservation and breeding studies due to the lack of full-length transcriptomic references. In this study, we generated 63.4 Gb of full-length transcriptome data from *M. guttatus*. After stringent quality filtering and redundancy removal, 32,647 full-length transcripts with an N50 of 2077 bp were obtained. Systematic comparison with published teleost’s full-length transcriptomes (Table 4) revealed our dataset demonstrates comparable quality metrics (isoform number and N50) to previous studies [23,36,37]. Notably, observed variations in assembly quality indices likely derive from two technical limitations: (1) the non-incorporation of complementary short RNA-seq sequencing data (e.g., Illumina platforms) for error correction, and (2) inherent biological variability in tissue sample integrity [38,39,40]. As the inaugural full-length transcriptomic resource for *M. guttatus*, this dataset establishes an indispensable genomic framework for evolutionary studies, stress adaptation research, and conservation-oriented molecular breeding initiatives.

Through systematic functional and structural annotation, 30,977 protein-coding genes were functionally annotated across public databases, and a large number of TFs, AS events, and SSRs were identified. Among the TFs, the zinc finger C2H2-type (Zf-C2H2) and basic helix–loop–helix (BHLH) families emerged as predominant groups. This is consistent with findings from most aquatic animals [46]. Zf-C2H2 proteins, constituting the largest TF class, demonstrate pleiotropic regulatory functions encompassing growth modulation, immune response coordination, and stress adaptation mechanisms [47]. Regrettably, this study was unable to further investigate the transcriptional regulation of HSP gene family members by TFs. The absence of 5′ untranslated regions (5′UTRs) in our full-length transcriptomic data precluded the prediction of TF binding sites within promoter regions, which are critical for elucidating regulatory networks. Furthermore, ethical and logistical constraints related to the conservation status of *M. guttatus* (Class II Protected Species in China) rendered stress experiments on wild-caught specimens unfeasible. In the future, we anticipate that high-resolution genome-wide sequencing data will enable comprehensive TF interaction mapping. Concurrently, controlled stress trials using artificial breeding *M. guttatus* populations could provide empirical validation of TF-mediated stress adaptation mechanisms. AS event enables a gene to generate multiple mRNAs that are translated into different proteins, thereby altering gene function [48]. A total of 918 AS events were identified, among RI events accounted for over 50%, which is similar to allied species *Ictalurus punctatus*, suggesting conserved splicing regulation mechanisms in *Siluriformes* [49]. It has been extensively demonstrated in other research that AS events can be harnessed to cope with various environmental stresses such as extreme temperature and high salinity [50,51]. Collectively, the identified TF and AS elucidate the transcriptional complexity of *M. guttatus*, establishing a molecular framework for investigating stress response mechanisms in this ecologically vulnerable species. These findings provide critical insights into the genomic adaptations underlying environmental resilience while furnishing essential resources for subsequent mechanistic studies. SSRs, as co-dominant genetic markers characterized by high polymorphism, excellent reproducibility, and good specificity, play important roles in analyzing genetic diversity, comparing kinship, and constructing genetic maps [52]. In this study, 11,380 SSRs were identified. It provides fundamental information for studying genetic diversity and support for its molecular marker-assisted breeding.

Long-term anthropogenic and environmental pressures have driven a severe population decline in *M. guttatus*. However, even artificially bred populations exhibit stringent environmental requirements and elevated mortality rates, suggesting inherent deficiencies in stress tolerance mechanisms. To address this, we systematically characterized the HSP superfamily in *M. guttatus*, identifying 93 hsp genes classified into HSP70 and HSP90 subfamilies [53]. Both HSP70 and HSP90 subgroups function as essential molecular chaperones critical for maintaining cellular proteostasis under stress conditions [54]. The HSP70 subfamily facilitates protein folding and assembly, thereby regulating enzymatic activity and substrate specificity [55]. Additionally, HSP70 isoforms modulate apoptotic pathways through dynamic interactions with key signaling proteins such as Bcl-2 family members and caspases [56]. The HSP90 subfamily specializes in conformational repair of misfolded proteins while orchestrating stress-responsive signaling cascades [57]. Beyond chaperone activity, HSP90 interacts with cell cycle regulators (e.g., CDKs) and transcriptional cofactors to mediate chromatin remodeling and gene expression modulation [58]. In previous studies, it was discovered that HSP primarily performs functions in the cytoplasm, explaining that the subcellular localization of most HSP70 and HSP90 is in the cytoplasm [53]. Notably, post-translational modification profiling identified limited glycosylation sites (none or a single) across most HSP isoforms. A notable exception was *MgHsp70-50*, which harbors five putative glycosylation sites. This anomaly may reflect stress-induced post-translational regulation, as glycosylation dynamics are known to influence pathogen resistance in teleost [59]. The unique glycosylation signature of *MgHsp70-50* warrants targeted investigation to elucidate its role in stress adaptation and immune modulation.

To elucidate evolutionary relationships among teleost species, we constructed a maximum likelihood phylogenetic tree of *hsp* family genes. The analysis revealed nine distinct clades comprising homologous genes, with limited cross-species divergence observed across fish lineages, suggesting conserved gene evolution. Notably, three genes (*hsp90aa1.1*, *hspa12a*, *and hspb12b*) were absent from the phylogenetic topology [60,61], a finding corroborated by their absence in protein–protein interaction networks. This pattern aligns with documented gene loss events in diverse ichthyofauna including *Oncorhynchus mykiss* [62], *Pleuronectiformes* [63], and *Sinocyclocheilus* [64]. Selective pressure analysis of 13 genes from protein network-identified orthologs shared with *Danio rerio* demonstrated significant purifying selection acting on the HSP family, concurrent with gene loss events [65]. These evolutionary mechanisms (purifying selection and gene loss) represent fundamental drivers of adaptation across metazoans [66], enabling functional specialization through genomic streamlining [67,68]. However, such historical adaptive strategies may confer reduced resilience against contemporary anthropogenic stressors. A parallel evolutionary trajectory is observed in koala (*Phascolarctos cinereus*) microbiomes, where Synergistaceae symbionts underwent gene loss in protein metabolism pathways to optimize eucalyptus leaf digestion [69]. While this specialization minimized nutritional competition, it created critical vulnerability to habitat perturbations. Catastrophic population declines followed Australia’s 2019–2020 bushfires that devastated eucalyptus ecosystems [70], culminating in the species’ threatened status designation [71]. Similar evolutionary pressures likely compounded the decline of *M. guttatus*. Initially driven by anthropogenic and environmental disruptions, population collapse was further exacerbated by genomic erosion, leaving surviving individuals increasingly vulnerable. These dual stressors collectively explain the species’ rapid demographic contraction. Our data reveal patterns of hsp family gene loss under purifying selection, analogous to evolutionary constraints observed in other threatened taxa. While genomic streamlining like gene loss may have historically enhanced fitness under stable conditions, it now limits adaptive capacity to modern stressors such as thermal fluctuations and pollution, potentially perpetuating the species’ downward trajectory. Notably, a significant positive selection gene *hsp70-4* (Ka/Ks = 1.69) was identified, strongly suggesting adaptive evolution under thermal stress regimes. This gene likely functions as a molecular chaperone critical for maintaining proteostasis during transient stress events, such as daily temperature spikes or hypoxic pulses, thereby enhancing survival in fluctuating habitats. The elevated Ka/Ks ratio indicates accelerated amino acid substitutions in its substrate-binding domain, potentially optimizing thermal stability and substrate recognition under proteotoxic conditions. These findings underscore *hsp70-4*’s dual role in stress sensing and adaptive plasticity.

Simultaneously, we have observed the complex interplay between HSP70 and HSP90 within the protein interaction network (Figure 6A). This was also observed in two other aquatic animals, *Ruditapes philippinarum* [72] and *Ciona Savignyi* [73]. To gain more insight into this relationship, exogenous proteins were introduced into the protein interaction network. The result showed that HOP interacts with most HSP70 and HSP90 proteins (Figure 6B). In previous studies, it has been discovered that HSP70 and HSP90 exhibit stronger interactions with HOP and form an HSP70-HOP-HSP90 complex. This complex can bind to various cytokines, demonstrating strong stress resistance and maintaining cellular homeostasis across a wide range of conditions [74,75,76]. In previous studies, HSP70 and HSP90 can form the complex HSP70-HOP-HSP90 with HOP, which further binds to the inactive Glucocorticoid receptor (GR) [77]. This ATP-dependent process ultimately yields activated GR complexes capable of nuclear translocation [78]. This is a complete molecular mechanism of HSP70-HOP-HSP90-dependent client protein remodeling, and this mechanism ensures the activity of many receptors’ HSP70-HSP90 interactions have been widely observed in fungi, plants, and mammals in the past [79,80], yet the study of this mechanism remains absent in fish. Given ethical and regulatory constraints prohibiting experimental manipulation of *M. guttatus*, we leveraged AlphaFold3—a deep learning framework for atomic-level protein structure prediction—to model the HSP70-HOP-HSP90 protein complex (Figure 6C and Table S2). Our study suggests that the HSP70-HOP-HSP90 interaction also exists and plays a crucial role in the stress response in *M. guttatus*. It deserves further investigation to provide more insight into improving the stress resistance of this endangered animal.

While this study provides novel insights into the stress response mechanisms of *M. guttatus* through comprehensive transcriptomic and evolutionary analyses, several limitations warrant careful consideration. At the same time, we have characterized the Hsp90 family’s molecular features and evolutionary history, but their role in mediating adaptive traits like temperature resilience remains speculative without direct ecological data. First, the sample size, though substantial for a non-model species, may not fully capture the genetic diversity across its natural range, potentially limiting the generalizability of findings to understudied populations or distinct ecological niches. Second, the transcriptomic data, generated via PacBio sequencing, lack complementary short-read RNA-seq data for error correction, which might affect the accuracy of splice variant annotations and low-abundance transcript detection. Additionally, the absence of functional validation experiments, such as CRISPR-based gene knockouts or protein interaction assays, leaves the biological significance of identified HSP variants and their regulatory networks partially speculative. Addressing these limitations through expanded field sampling, multi-omics integration, and experimental validations will be essential for refining stress response models and informing conservation strategies.

## 5. Conclusions

Our study has successfully sequenced and analyzed the full-length transcriptome of *M. guttatus*, generating 32,647 high-quality transcripts (N50 = 2077 bp). Functional annotation revealed 30,977 protein-coding genes, with 11,830 SSRs and 918 AS events (62% RI-type), highlighting transcriptional complexity and genetic diversity. Dominant transcription factors (e.g., Zf-C2H2, bHLH) and stress-responsive AS patterns align with mechanisms observed in allied teleosts, suggesting conserved regulatory networks for environmental adaptation. Based on the full-length transcriptome, a substantial amount of data related to gene structure and function was derived, laying a solid foundation for improving the current genome assembly and facilitating future transcriptome annotation. Comprehensive analysis of the HSP gene family revealed 93 hsp genes, with phylogenetic and selective pressure analyses indicating gene loss and purifying selection. This genomic streamlining, which may be historically advantageous, may have reduced stress resilience under modern environmental challenges (e.g., thermal fluctuations, pollution), potentially driving population decline. To address this, we characterized key adaptive mechanisms, including positively selected HSP variants, HSPs with multiple glycosylation sites (e.g., *MgHsp70-50*), and the evolutionarily conserved HSP70-HOP-HSP90 interaction network. These findings provide critical insights for optimizing *M. guttatus* stress tolerance through genomic-assisted breeding strategies. The high-quality transcriptome data also advance genome assembly refinement and transcriptome annotation, establishing a foundation for future functional studies and conservation efforts.

## Figures and Tables

**Table 1:** The statistics of polished isoforms reads.

Data	Number
Total number	32,647
Total length (bp)	58,217,443
Maximum Length (bp)	8049
Minimum Length (bp)	54
Average Length (bp)	1783
N50 Length	2077

**Table 2:** Scores of molecular dockings.

Group of Protein	Docking Score	Confidence Score	Ligand RMSD
HSP70-HSP90	−227.49	0.8249	27.59
HOP-GR	−242.92	0.8651	78.01
HSP70-HSP90-HOP-GR	−229.15	0.8296	37.02

**Table 3:** Selective pressure analysis of the *hsp* gene family.

*M. guttatus* Gene	*D. rerio* Gene	Ka	Ks	Ka_Ks	Selection
*MgHsp90-21*	*trap1*	0.11933	1.416211	0.08426	Purify
*MgHsp70-59*	*hyou1*	0.037815	1.147255	0.032961	Purify
*MgHsp70-29*	*hspa9*	0.072756	1.685386	0.043169	Purify
*MgHsp70-10*	*hspa8b*	0.155257	1.116471	0.13906	Purify
*MgHsp70-63*	*hspa8*	2.510801	1.483283	1.692732	Positive
*MgHsp70-31*	*hspa4a*	0.087347	1.249427	0.06991	Purify
*MgHsp70-34*	*hspa14*	0.071319	1.10135	0.064756	Purify
*MgHsp70-45*	*hspa13*	0.112163	1.836793	0.061065	Purify
*MgHsp90-26*	*hsp90b1*	0.045808	1.159956	0.039491	Purify
*MgHsp90-28*	*hsp90ab1*	0.031203	1.340752	0.023273	Purify
*MgHsp90-29*	*hsp90aa1.2*	0.023897	2.474259	0.009658	Purify
*MgHsp70-24*	*hsp70.2*	0.03791	0.880748	0.043043	Purify
*MgHsp70-53*	*hspa5*	0.03482	1.330795	0.026165	Purify

Tips: Ka (Non-synonymous Substitution Rate): It measures the frequency of non-synonymous mutations in the coding region of a gene, which affects the amino acid sequence and function of the protein; Ks (Synonymous Substitution Rate): It measures the frequency of synonymous mutations in the coding region of a gene and does not affect the amino acid sequence of the protein. Ka/Ks > 1 indicates positive evolution, and Ka/Ks < 1 indicates purified selection.

**Table 4:** Full-length transcriptome data obtained in fish.

Fish Species	Data Sources	Isoform Number	N50 Length	References
*Mystus guttatus*	PacBio	32,647	2077	This paper
*Gymnocypris namensis*	PacBio	125,396	2044	[41]
*Hexagrammos otakii*	PacBio and Illumina	42,225	2482	[42]
*Acipenser dabryanus*	PacBio and Illumina	155,348	3365	[43]
*Hypophthalmichthys nobilis*	PacBio, Illumina and Reference genome	63,873	1741	[44]
*Atractosteus tropicus*	PacBio and Illumina	80,065	1664	[45]

## Data Availability

The data presented in this study are available on request from the corresponding author due to confirm the species in this study is a second-class protected animal in China.

## Supplementary Materials

The following supporting information can be downloaded at: https://www.mdpi.com/article/10.3390/biology14070840/s1, Table S1: Physicochemical properties, subcellular localisation and glycosylation sites of the *MgHSP*; Table S2: Detailed information of the molecular docking models of the three complexes HSP70-HSP90/HOP-GR/HSP70-HOP-HSP90-GR.
